# A novel strain of *Bacteroides fragilis* enhances phagocytosis and polarises M1 macrophages

**DOI:** 10.1038/srep29401

**Published:** 2016-07-06

**Authors:** Huimin Deng, Zhengchao Li, Yafang Tan, Zhaobiao Guo, Yangyang Liu, Ye Wang, Yuan Yuan, Ruifu Yang, Yujing Bi, Yang Bai, Fachao Zhi

**Affiliations:** 1 Guangdong Provincial Key Laboratory of Gastroenterology, Inst. of Gastroenterology of Guangdong Province, Department of Gastroenterology, Nanfang Hospital, Southern Medical University, Guangzhou 510515, China; 2 State Key Laboratory of Pathogen and Biosecurity, Beijing Institute of Microbiology and Epidemiology, No. 20 Dongdajie, Fengtai District, Beijing 100071, China; 3 Guangzhou ZhiYi biotechnology Co. Ltd. No. 3, Lanyue Road, International Business Incubator Building F, Guangzhou 510515, China

## Abstract

Commensal *Bacteroides fragilis* possesses immune-regulatory characteristics. Consequently, it has been proposed as a potential novel probiotic because of its therapeutic effects on immune imbalance, mental disorders and inflammatory diseases. Macrophages play a central role in the immune response, developing either a classical-M1 or an alternative-M2 phenotype after stimulation with various signals. The interactions between macrophages and *B. fragilis*, however, remain to be defined. Here, a new isolate of *B. fragilis*, ZY-312, was shown to possess admirable properties, including tolerance to simulated gastric fluid, intestinal fluid and ox bile, and good safety (MOI = 100, 200) and adherent ability (MOI = 100) to LoVo cells. Isolate ZY-312 cell lysate promoted phagocytosis of fluorescent microspheres and pathogenic bacteria in bone marrow-derived macrophage (BMDM) cells. Gene expression of IL-12, iNOS and IL-1β in BMDM cells was increased after treatment with ZY-312, indicating the induction of M1 macrophages, consistent with enhanced secretion of NO. Cell surface expression of CD80 and CD86 was also increased. This study is the first to demonstrate that *B. fragilis* enhances the phagocytic functions of macrophages, polarising them to an M1 phenotype. Our findings provide insight into the close relationship between *B. fragilis* and the innate immune system.

Commensal bacteria reside in the gastrointestinal tract and affect host health both locally and systemically^1^. The relationship between these symbiotic bacteria and their host is far more complex than simple coexistence with nutritional benefits^2^. Recently, the more obscure contributions of symbiotic bacteria to host health have been uncovered and the “hygiene hypothesis”, which states that exposure to commensal bacteria is necessary to protect the host from unrelated immune diseases, has been proposed and debated^3^. *Bacteroides fragilis*, is a ubiquitous Gram-negative obligate anaerobe that colonises the lower gut of mammals, and *Bacteroides* species constitute a numerically prominent part of the gut flora^4^. Despite comprising 1% or less of *Bacteroides* sp., *B. fragilis* accounts for more than 50% of anaerobic infections in humans, which predominantly lead to intra-abdominal abscesses^5^. *B. fragilis* was initially considered to be an opportunistic pathogen; however, it is now recognised to exhibit both pathogenic and beneficial characteristics in the host^6^. *B. fragilis* has been shown to exert powerful immunoregulatory benefits predominantly mediated by polysaccharide A (PSA), a zwitterionic polysaccharide, shown to protect animals against various inflammatory diseases such as trinitro-benzene-sulfonic-sulfonic acid and *Helicobacter hepaticus*-induced colitis^7^. Another report demonstrated that application of *B. fragilis* relieved ASD symptoms in mice, exhibiting considerable probiotic potential^8^.

Much research has been performed to explore the latent mechanisms involved in the immune-regulatory effects of *B. fragilis*. However, these studies have mainly focused on dendritic cells^9^,^10^ and the adaptive immune system^7^, the effects of *B. fragilis* on macrophages of the innate immune system have yet to be fully explored. Macrophages form an integral part of the innate immune system, presenting foreign antigens, mediating early pathogen control and coordinating downstream immune responses^11^,^12^. When infection occurs, macrophages assemble and phagocytise pathogens, leading to direct microbiocidal responses^13^ or antigen presentation to T cells activating adaptive immune responses^14^. Furthermore, cytokines secreted by macrophages mediate a wide range of immunoregulatory effects^14^,^15^. For example, IL-12 produced by macrophages mainly boosts activation of Th1 cells conferring cellular immunity, while IL-10 activates Th2 cells and suppresses cellular immune responses. To better understand the opposing roles of macrophages, the M1/M2 paradigm was proposed, which correlates to Th1/Th2 responses^16^. The M1/M2 paradigm provides a framework with which to distinguish the states and functions of macrophages, with M1 and M2 macrophages at either end of a continuum and the intermediate states exhibiting overlapping functions^15^. The main distinguishing feature is that M1 macrophages metabolise arginine to NO, whereas M2 macrophages produce ornithine^16^. Furthermore, M1 macrophages are associated with IL-12 and IL-8/CCL production, cell surface expression of CD80/CD86^14^, promoting Th1 responses and possess strong microbiocidal and tumoricidal activity^17^. By contrast, M2 macrophages are linked with IL-10 production, promoting Th2 responses and aiding tissue repair, as well as ameliorating inflammation^14^,^15^.

In this study, we isolated a novel bacterial strain from the faeces of a healthy baby. After morphological and genetic analysis, this strain was confirmed to belong to *B. fragilis* and was designated ZY-312. The probiotic properties of this strain were studied, focusing on the effects on macrophages using an *in vitro* model of bone marrow-derived macrophages (BMDM). Our findings indicated that a cell lysate of ZY-312 significantly enhanced the phagocytic functions of BMDM, both on fluorescence beads and pathogens. Increased gene expression of IL-12, IL-1β and iNOS, as well as the production of NO and cell surface expression of CD86 and CD80, were also detected. We established for the first time that *B. fragilis* enhances the function of macrophages, polarising them towards the M1 type.

## Results

### Isolation and molecular identification of ZY-312

**Figure 1:**
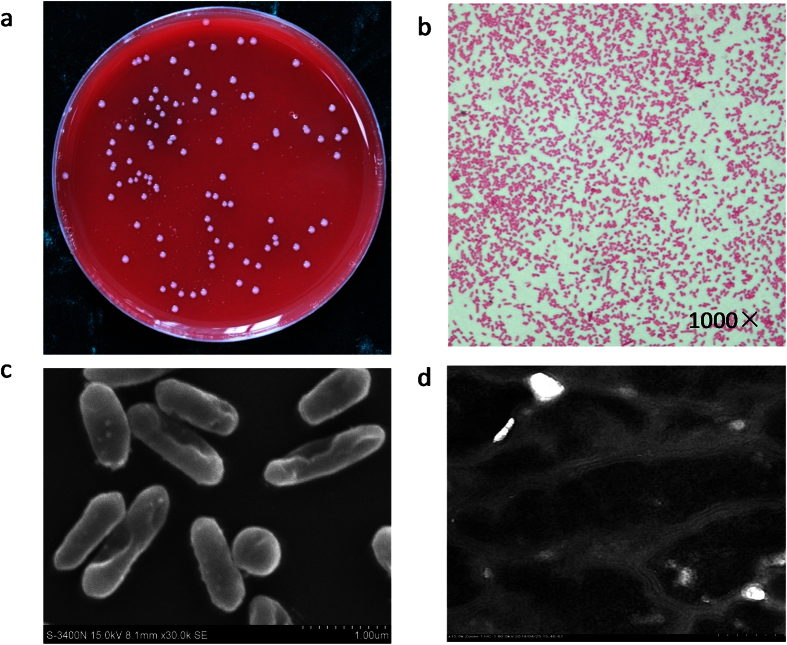
Morphological characteristics of ZY-312. (**a**) Colonies on TSA (5% sheep blood) agar cultured anaerobically after 48 h at 37 °C. (**b**) Observations under an optical microscope after Gram-staining. (**c**) Observations under a scanning electron microscope. (**d**) Observations under a transmission electron microscope.

The bacterial strain ZY-312 isolated from the faeces of a healthy breast-fed infant was found to be similar to *B. fragilis* both morphologically and in terms of its growth characteristics, which is shown in Fig. 1a–d. By Gram staining and direct observation by scanning electrochemical microscopy and transmission electron microscopy, this strain was found to be Gram-negative, and rod shaped with rounded ends. The physiological and biochemical features of this strain were also in accordance with *B. fragilis* as described in Bergey’s manual. The results of 16S rRNA gene sequencing demonstrated that the nucleotide sequence of this strain was up to 99% identical to that of type stain *B. fragilis* ATCC 25285 (GenBank database), and showed high similarity to JCM11019, confirming that the isolate was a strain of *B. fragilis*.

### Growth characteristics of ZY-312

**Figure 2:**
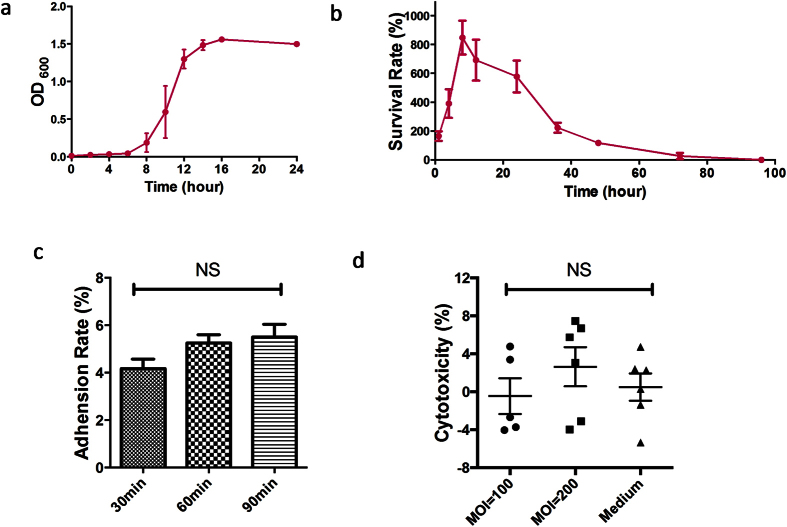
Fundamental properties of ZY-312. (**a**) Growth curve under anaerobic culture for 24 h. (**b**) Survival rate after exposure to air for the bacteria grown in TSB (10% FBS) for 96 h (mean ± SE; N = 3). (**c**) Adhesion rate of ZY-312 to LoVo cells after 30, 60 and 90 min at a MOI = 100. (**d**) Cytotoxicity to LoVo cells at a ratio of infection of 100:1 and 200:1 after 4 h. Culture medium was used as a negative control (mean ± SE; N = 3, ANOVA).

The results of growth kinetic studies of ZY-312 in TSB supplemented with 10% FBS (v/v), grown under anaerobic culture conditions, are shown in Fig. 2a. Strain ZY-312 entered exponential phase after 8 h, and progressed to stationary phase at around 16 h. Throughout our study, we selected late logarithmic phase (12–16 h) cells because of the peak in the number and viability of bacteria.

### Tolerance to air

As most assays are performed aerobically, we tested the viability of ZY-312 after exposure to air, and the survival rates are presented in Fig. 2b. Isolate ZY-312 showed resistance to air for at least three days, which agreed with a previous report^18^. Viable counts of ZY-312 were increased more than 8-fold at 12 h, then survival rates gradually decreased with few surviving cells at 72 h. This demonstrated that ZY-312 is not a strictly anaerobic bacterium, and sufficiently air resistant for the *in vitro* assays performed in this study.

### Tolerance to stimulated digestive fluid

To investigate the ability of ZY-312 to tolerate digestive juice, an *in vitro* test was adopted to test the survival rate after exposure to simulated gastric fluid (SGF), simulated intestinal fluid (SIF) and bile under different conditions (pH or concentrations) and time points. ZY-312 resistance levels to SGF an SIF are shown in Table 1a. In this study, ZY-312 was unable to survive on exposure to SGF of pH 2.0 (data not shown), but survived well with SGF at pH 3.0 or pH 4.0, and with SIF at pH 6.8. The survival ability of ZY-312 in ox bile is presented in Table 1b. ZY-312 showed high resistance to bile, which was consistent with a previous study^19^ that reported that *B. fragilis* showed good tolerance to bile salt.

### Adhesion to colon cancer cells

The results of an adhesion assay to assess ZY-312 (MOI = 100) adhesion to LoVo cells are shown in Fig. 2c. Differences in the adhesion rate between each time point were not significant (*p* > 0.05), but the adhesion rate showed a steady increase over time reaching 4.18 ± 1.18, 5.25 ± 1.03 and 5.51 ± 1.60% of the adhesion activity to LoVo cells at 30, 60 and 90 min, respectively. This result demonstrated the adherent ability of ZY-312 to colon cells *in vitro*.

### Cytotoxicity of viable ZY-312

The results of an assay to assess ZY-312 cell cytotoxicity to LoVo cells are presented in Fig. 2d. In this assay, LoVo cells were infected with intact bacteria at different infection ratios (MOI = 100, 200) for 4 h. LDH released by the damaged cells in the supernatant was measured to estimate cytotoxicity. The levels of cytotoxicity were found to be 0.50 ± 3.53, −0.46 ± 4.20 and 2.64 ± 5.03% for the control, and the 100:1 and 200:1 infection ratio groups respectively. No significant differences (*p* > 0.05) were found between the groups, suggesting that intact viable ZY-312 cells were not cytotoxic *in vitro*.

### Enhancing the phagocytic functions of BMDM cells

**Figure 3:**
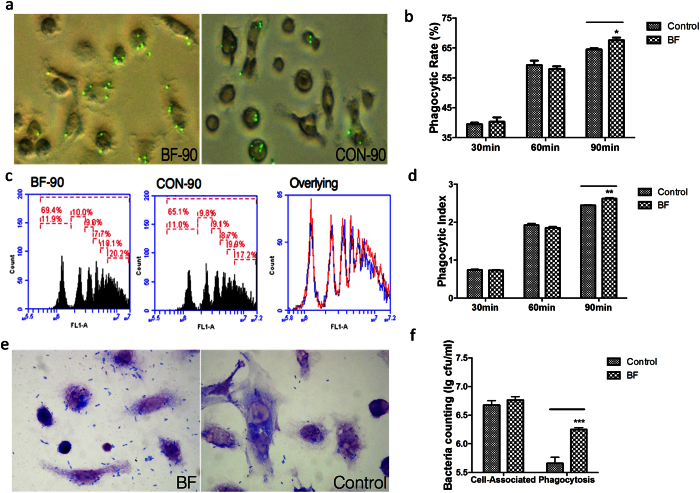
*In vitro* phagocytosis activity of BMDM cells modulated by the cell lysate of ZY-312 (Initial MOI = 500). (**a**) Fluorescent microspheres swallowed for 90 min by BMDM cells treated with the ZY-312 cell lysate. (**b**) Phagocytic rate of swallowing of fluorescent beads by BMDM cells after 30, 60 and 90 min for ZY-312-treated cells vs. control cells. (**c**) Histograms showing beads phagocytised by BMDM cells, which can be distinguished by peak area and mean fluorescence intensity (MFI). ZY-312 treated (left) vs. untreated (middle) and the overlying histograms (right) for treated (red) and untreated (blue).(**d**) Phagocytic index was calculated according to the number of beads phagocytised by each group of BMDM cells for 30, 60 and 90 min. (**e**) EHEC phagocytised (MOI = 50) for 30 min, cell lysate-treated group (left), untreated group (right). (**f**) Cell associated-bacteria and phagocytic bacteria in phagocytosis assay (mean ± SE; N = 3; *p < 0.05, **p < 0.01, ***p < 0.001, t test).

To explore whether ZY-312 affected BMDM phagocytic function, fluorescence microspheres and EHEC were used as foreign entities in a phagocytosis assay. The results with fluorescent beads (Fig. 3a–d) demonstrated that the ZY-312 cell lysate (initial MOI = 500) not only promoted more BMDM cells to phagocytose beads (*p* < 0.05), but also encouraged BMDM to phagocytose more beads (*p* < 0.01), with significant differences observed between the treated and untreated group at 90 min. In the pathogen phagocytosis assay, we infected BMDM cells with EHEC (MOI = 50). Accordingly, ZY-312 cell lysate had no influence on BMDM cell-associated bacteria (*p* > 0.05), but improved the phagocytosis of EHEC significantly (*p* < 0.001) (Fig. 3e,f). These findings indicated that ZY-312 cell lysate modulate and facilitate BMDM cells to phagocytose foreign entities.

### Polarising BMDM cells to become M1-like macrophages

**Figure 4:**
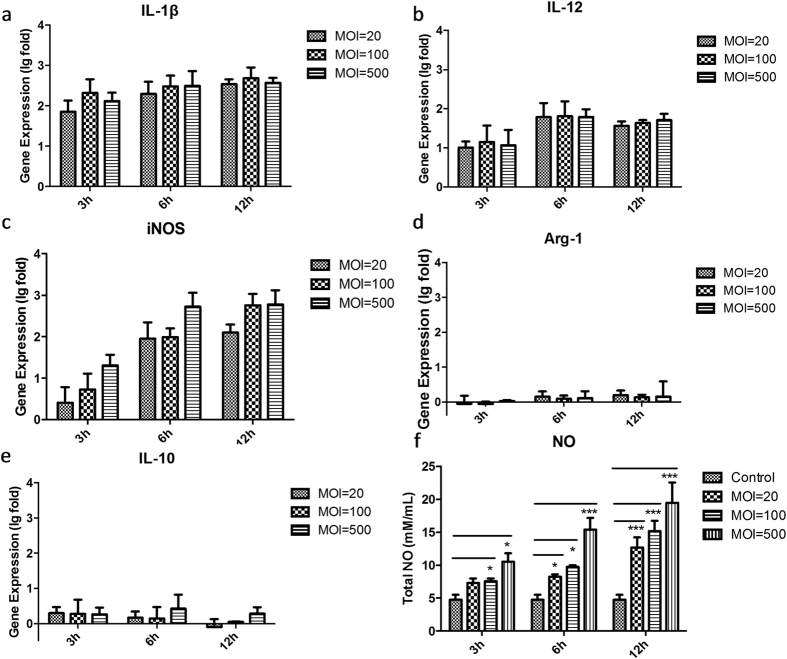
Change in M1/M2 ratio, specific marker gene expression and total NO release by BMDM cells stimulated with different inoculum ratios of ZY-312 cell lysate for 3, 6 and 12 h. Gene expression of M1 related markers: IL-1β (**a**), IL-12 (**b**) and specific marker iNOS (**c**), and M2 related gene markers: specific marker Arg-1 (**d**) and IL-10 (**e**), as detected by RT-PCR. The data are shown as log-fold changes after logarithm conversion. Total NO (**f**) released in the culture supernatant was detected by ELISA. (Mean ± SE; N ≥3; *p < 0.05, **p < 0.01, ***p < 0.001, One-way ANOVA, t test).

**Figure 5:**
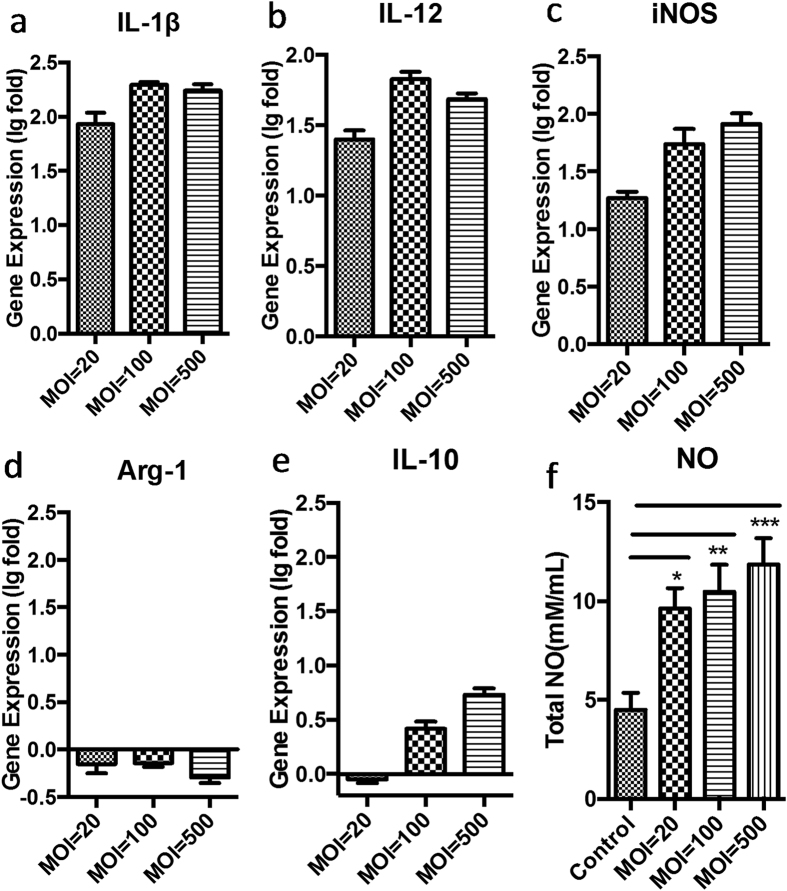
Change in M1/M2 ratio, specific marker gene expression and total NO release by BMDM cells infected with different infection ratios of ZY-312 viable bacteria for 4 h. Gene expression of M1 related markers: IL-1β (**a**), IL-12 (**b**) and specific marker iNOS (**c**), and M2 related gene markers: specific marker Arg-1 (**d**) and IL-10 (**e**), as detected by RT-PCR. The data are shown as log-fold changes after logarithm conversion. Total NO (**f**) released in the culture supernatant was detected by ELISA. (Mean ± SE; N ≥3; *p < 0.05, **p < 0.01, ***p < 0.001, One-way ANOVA, t test).

Gene analysis of M1/M2 related and specific markers expressed by BMDM cells treated by ZY312 cell lysate or ZY312 viable bacteria are shown in Figs 4a–e and 5a–e, respectively. After treatment with ZY-312 cell lysate, IL-1β expression at the transcriptional level in BMDM cells was increased regardless of the initial inoculum of bacteria from which the lysate was obtained, whereas IL-12 and iNOS expression increased in a time-dependent manner. Under the same conditions, IL-10 was expressed at a relatively low level while Arg-1 was unchanged or even down regulated. To further confirm such polarisation, we analysed the total amount of NO in the cell supernatant (Fig. 4f). Consistent with the transcriptional changes, the amount of NO secreted was significantly increased (*p* < 0.05) in a manner dependent on incubation time and the bacterial inoculum to obtain the lysate. Similar results were obtained in infection experiments with viable bacteria (Fig. 5a–f). All these results demonstrated that ZY-312 promotes IL-12, IL-1β and NO expression in BMDM cells, and polarises them to become M1-like macrophages.

### Enhancing cell surface antigen expression in BMDM cells

**Figure 6:**
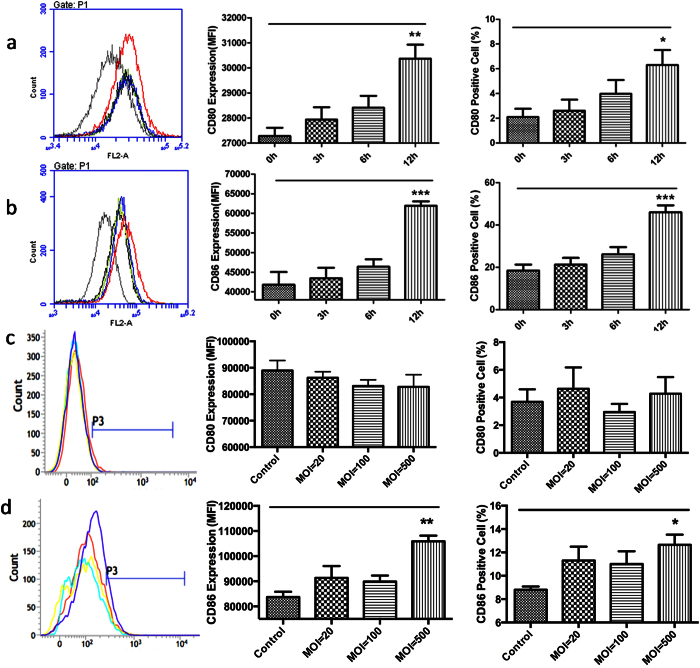
BMDM cell surface expression of CD80 and CD86 after treatment with ZY-312. Expression of CD80 and CD86 on the BMDM cell surface after exposure to ZY-312. (**a**,**b**) BMDM cells treated with ZY-312 cell lysate (initial MOI = 500) for 3, 6, 12 h. a-1. Overlay fluorescence from CD80. a-2. Mean fluorescence intensity (MFI) of CD80 expression. a-3. Ratio of CD80 positive cells; b-1. Overlay fluorescence from CD86. b-2. MFI of CD86 expression. b-3. Ratio of CD86 positive cells. (**c**,**d**) BMDM cells infected with ZY-312 (MOI = 20, 100, 500) for 4 h. c-1. Overlay fluorescence from CD80. c-2. Mean fluorescence intensity (MFI) of CD80 expression. c-3. Ratio of CD80 positive cells; d-1. Overlay fluorescence from CD86. d-2. MFI of CD86 expression. d-3. Ratio of CD86 positive cells.(Mean ± SE; N ≥3; *p < 0.05, **p < 0.01, ***p < 0.001, One-way ANOVA, t test).

The cell surface expression of CD86 on BMDM cells was significantly enhanced after treatment both with ZY-312 cell lysate and bacteria (Fig. 6b,d), while CD80 expression was enhanced only with cell lysate (Fig. 6a,c). Taking into consideration the nutrients exhaustion and growth kinetics of ZY-312 in air, 4 h was chosen as the end point for experiments with living bacteria while the effects of cell lysate were assessed over a time course up to 12 h. These experimental differences might explain the lack of CD80 induction by viable bacteria. In conclusion, ZY-312 cell lysate promoted BMDM cells to express CD80 and CD86 after treatment for 12 h, and this effect has been partially reproduced in experiments with viable bacteria (MOI = 500).

## Discussion

*Bacteroides fragilis*, a ubiquitous anaerobic bacterium that colonises the lower gut, was recently proposed as a potential probiotic because of its considerable clinical benefits on inflammatory and mental disorder diseases^6^,^8^. In this study, we isolated ZY-312 and confirmed that it is a strain of *B. fragilis* by morphological analysis and 16 s rRNA sequencing according to Bergey’s manual. First, we explored its fundamental properties, and demonstrated its high tolerance to SGF, SIF and bile, with the exception of low pH SGF (pH = 2.0). Given that our experiments were performed in air, we tested the survival rate of this isolate in air over time and found that it survived at least 72 h, a result that was consistent with previous reports^18^. Furthermore, we demonstrated the safety of this isolate *in vitro* at MOI = 200 and its adhesive ability to colon cells. Taken together, our findings indicate that ZY-312 is a new-isolated strain of *B. fragilis* with some desirable characteristics.

Much research has been performed on the interaction between *B. fragilis* and the innate immune system, especially for PSA. The reciprocity between DCs and *B. fragilis* has been explored comprehensively and some insight into the molecular mechanisms has been reported^9^,^10^,^20^. However, little is known regarding the relationship between *B. fragilis* and macrophages. Macrophages are the first line of defence against pathogens when microorganisms invade^11^, and function as the control centre during host defence^12^. Macrophages have been designated as M1 or M2 based on their activation of Th1 or Th2 responses, highlighting the crucial role of macrophages in the host immune system^16^. In this current study, we found that following exposure to the ZY-312 cell lysate for 12 h, BMDM cells phagocytosed more fluorescence beads and pathogens compared with the control group (Fig. 3), implying the enhanced phagocytic function of macrophages treated with *B. fragilis*. Furthermore, we observed significantly upregulated gene expression of IL-12 and IL-1β in BMDM cells after treatment with cell lysate or viable bacteria. Increased expression of the iNOS gene and release of total NO into the supernatant were also observed, confirming the results of a previous report^21^. It has been reported that purified PSA induced production of IL-12^22^ and NO^21^,^23^ by dendritic cells. DCs internalise PSA through the TLR-2 pathway and present it to T cells^23^, and this process is NO dependent since NO is necessary for the degradation of PSA. The expression of CD80 and CD86 were increased on BMDM cells (Fig. 6) after treatment, and similar results have previously been shown for DCs^21^. Exposure to PSA reportedly increases the expression of co-stimulatory molecules CD80 and CD86^1^,^24^ and signalling through CD80 and/or CD86 to activate the generation of CD4^+^ T cells^25^.

Interestingly, *B. fragilis* seems to play a complex role in interacting with the immune system, which is dependent on the microenvironment. Once *B. fragilis* (ATCC 25285) leaks into the abdominal cavity, it leads to inflammation due to the increased production of pro-inflammatory cytokines TNF-α and IL-1β and abscess formation, then PSA is internalised by antigen-presenting cells after antigen presentation loading on the MHC II compartment to generate IL-10 producing T cells for controlling excessive inflammation^6^,^26^,^27^. This process is mediated by the complex interaction between *B. fragilis* and the innate as well as adaptive immune system. *B. fragilis* appears to initially develop a M1-like response to induce antimicrobial molecules that assemble immune cells and promote microbicidal molecules to kill and confine other gut microorganisms to prevent a more serious infection^6^. Our finding that *B. fragilis* promotes IL-12 production and phagocytic functions in macrophages provides evidence for this. Then, increased expression of CD80 and CD86 and degraded PSA loading on the surface of antigen-presenting cells, results in the generation of IL-10-producing T cells that aid the transition to an M2-like response to avoid an excessive inflammation response^6^.

The role of *B. fragilis* has recently been reconsidered and it is now thought that the development of an abscess may not be harmful to the host but may protect it from serious or even life-threatening complications^6^. Moreover, *B. fragilis* has been proposed as a potential probiotic because of the profound beneficial impacts of this organism on allergic and mental disorder diseases. However, much work is still needed to further elucidate the intricate and intimate relationship between *B. fragilis* and the host immune system. Our study is the first to systemically study the immune regulatory effects exerted by *B. fragilis* on macrophages *in vitro*, and to demonstrate that *B. fragilis* not only enhances the phagocytic functions of macrophages, but also polarises them to an M1 phenotype, providing new insight into our understanding of potential probiotic characteristics of *B. fragilis*.

## Methods

### Isolation of strain ZY-312 and molecular identification

The bacterial strain ZY-312 was isolated from the faeces of a healthy, breast-fed, infant as reported previously^28^. The collection of stool sample was part of a study approved by the Medical Ethics Committee of NanFang Hospital (NFEC-2014-040) and the procedure was performed in accordance to Institutional Review Board guidelines. A consent form was read and signed by parents of that baby. The isolated strain presented morphological and biological characteristics similar to those of *B. fragilis*, as described in Bergey’s manual. Genomic DNA from the strain was extracted using a DNA extraction kit (Qiagen) and 16S rRNA gene amplification was performed by PCR using the universal primers 27 F (5′-AGAGTTTGATCCTGGCTCAG-3′) and 1492 R (5′-GGTTACCTTGTTACGACTT-3′) (Lanes D–J). Amplified products were sequenced (Biomed) and the BLAST algorithm was used to search for homologous sequences in the NCBI database.

### Growth curve and air exposure

Strain ZY-312 was cultured in sterile tubes containing 10 ml of tryptone soy broth (TSB) supplemented with 5% fetal bovine serum (FBS), grown anaerobically at 37 °C in anaerobic glove box (Bugbox, Ruskin) for 24 h. Optical density at 600 nm was detected every 2 h. Given that most of the following tests were performed in air, we first verified the resistance of the strain to the atmosphere. Strain ZY-312 in late log phase was harvested and suspended in TSB (plus 5% FBS) to 10^9^ cfu/ml, incubated aerobically at 37 °C for 96 h. The survival was determined by the spread counting.

### Simulated gastric fluid, intestinal fluid and bile tolerance assay

Tolerance assays were performed as previously reported^29^,^30^. For preparation of SGF and SIF, 1 g/L pepsin (Sigma) or 10 g/L trypsin enzyme (Sigma) was supplemented with PBS, respectively. Using 1 M sodium hydroxide (Sinopharm) and 2.75 M hydrochloric acid solution (Sinopharm), SGF was adjusted to pH 2.0, 3.0, 4.0, and SIF to pH 6.8. Bacterial cells harvested after 14 h incubation (late exponential phase) were collected by centrifugation at 2000 g for 5 min at RT, washed twice with PBS and suspended to 10^9^ cfu/ml in SGF or SIF. Cell suspensions were then incubated at 37 °C anaerobically for 0, 1, 2 or 3 h. The viability of samples was determined by the spread plate method. Similarly, bacterial cells were collected as described above and adjusted to 10^−4^, 10^−6^ or 10^−8^ cfu/ml in bile containing 0, 1, 2 or 4% ox gall bladder powder (Sangon) in sterile TSB (plus 5% FBS). The mixture was incubated at 37 °C anaerobically for 0, 1, 2 or 4 h and each samples was serially diluted and spread on agar. The survival rate was determined by the ratio between the counts of viable bacteria and initial bacteria.

### Adhesion assay

LoVo cells were grown in DMEM/F12 (1:1) medium (Gibco) supplemented with 10% heat-inactivated FBS (MP); penicillin (100 U/ml), and streptomycin (100 ng/ml) in an incubator with 95% (v/v) humidified air and 5% (v/v) CO_2_ at 37 °C. LoVo cells were seeded at 1 × 10^5^ cells/well in 24-well plates overnight. Bacterial cells were harvested and adjusted to 1 × 10^7^ cfu/ml (multiplicity of infection, MOI = 100) with cell culture medium, co-incubated with LoVo cells at 37 °C for 30, 60 or 90 min. After incubation, non-adhered bacteria were removed by washing 3 times with PBS. The adherent bacteria were detached with 0.5 ml 0.25% trypsin (Gibco) and suspended in 0.5 ml PBS. The adherent ZY-312 cells were serially diluted and spread on TSA agar plates.

### Cytotoxicity against colon cells

The *in vitro* safety of ZY-312 was tested on LoVo cells using a Cytotoxicity Detection Kit^Plus^ (LDH) (Roche). LoVo cells were seeded at 5 × 10^4^ cells per well into 96-well plates and incubated overnight. 100 μl bacteria suspension (5 × 10^7^ or 1 × 10^8^ cfu/ml, MOI = 100 or 200) was added per well. After co-incubation at 37 °C in 5% (v/v) CO_2_ and 95% (v/v) humidified air for 4 h, absorbance values were determined using a microplate reader (SpectraMax, M2) and cytotoxicity was calculated with the following equation provided by the manufacturer’s protocol.

### BMDM isolation

Bone marrow-derived macrophage (BMDM) cells from mice were stimulated with recombinant mouse M-CSF (R&D) as reported previously^21^. Briefly, the bone marrow cells were stimulated with 20 ng/ml M-CSF. After 7 days culture, more than 90% pure BMDM were obtained. For the following tests, BMDM cells were harvested using a cell scraper (Costar) and cultured at 5 × 10^5^ cells/well in a fresh 24-well plates overnight.

### Preparation of ZY-312 cell lysate

ZY-312 cell lysates were prepared in advance should be used immediately. ZY-312 cell wall were broke by repeated ultrasonic sonication (Branson) on ice at 50 W for 10-s, at 10-s intervals, until cells were completely ruptured. The efficiency of cell breakage was confirmed by TSA agar plate counting before and after sonication. Centrifugation at 8000 g at 4 °C for 10 min and filtering were performed to ensure bacteria-free.

### Fluorescence phagocytosis assay

Fluorescent microspheres (Molecular Probes, 1.0 μm, carboxylate-modified) were pretreated and adjusted with culture medium to about 2 × 10^9^ microspheres/ml. Bath sonication for 30 min immediately before the phagocytosis assay was necessary to disperse the beads. After cell lysate (initial MOI = 500) treatment for 12 h, replaced 300 μl of beads to each well (using culture medium as a negative control), incubated at 37 °C for 30, 60 or 90 min. Cells were washed with cool PBS three times and collected, detected and analysed by flow cytometry (BD, Accuri, C6). Nonspecific adherent beads were not considered as microspheres and were due to low nonspecific binding. Phagocytosis rate and phagocytosis index were calculated using the following equations:


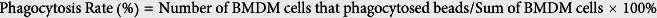



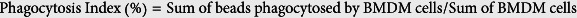


where, the Sum of beads phagocytosed by BMDM cells = Cells that phagocytosed 1 bead ×1 + Cells that phagocytosed 2 beads ×2 + Cells that phagocytosed 3 beads ×3 + Cells that phagocytosed 4 beads ×4 + Cells that phagocytosed 5 beads ×5 + Cells that phagocytosed more than 5 beads ×6. The numbers of beads phagocytosed was determined by overlying the fluorescence value and peak area with the interval.

### Pathogen phagocytosis assay

The pathogen used for this study was *Enterohaemorrhagic Escherichia coli* (EHEC) (Lab stock, No. CBSLAM00087), a serotype of *E. coli* accounts for haemorrhagic diarrhoea and kidney failure^32^. A pathogen phagocytic assay was performed according to a previous report^33^. EHEC cells were harvested from overnight culture in LB and suspended in DMEM at 2 × 10^7^ cfu/ml. 0.5 ml EHEC suspension were added to BMDM cells treated with cell lysate (initial MOI = 500) for 12 h, incubated at 37 °C for 30 min. To detect cell-associated bacteria (including both adherent and phagocytosed bacteria) after incubation, cells were washed three times with cool PBS, and 0.5 ml per well of 0.1% TritonX-100 solution (Sigma) was added for 3 min to lyse cells (3 min was sufficient for total lysis under these conditions). Additionally, at same time point, the supernatant was replaced with culture medium containing 50 μg/ml gentamycin and incubated for 30 min to kill all extracellular bacteria. Cells were washed, lysed as above, and bacteria were counted after serial dilution and plating on LB agar.

### RT-PCR assays

BMDM cells (six replicated wells per group) were treated with ZY-312 cell lysates (initial MOI = 20, 100, 500) for 3, 6, 12 h or ZY-312 viable bacteria (MOI = 20, 100, 500) for 4 h, and total RNA was extracted using the Purelink^TM^ RNA kit (Ambion) following the manufacturer’s protocol. The concentration and quality of the extracted RNA was determined by spectrophotometric measurements at OD_260_ (ND-1000; NanoDrop). Isolated total RNA (5 μg) was transcribed to cDNA using Superscript II reverse transcriptase (Invitrogen) and oligo (dT) primers (Invitrogen) following the manufacturer’s protocol. After purification with the QIAquik PCR purification kit (Qiagen), purified cDNA was used as template in qRT-PCR assays. Quantitation RT-PCR was performed for IL-10, IL-12 IL-1β, iNOs, Arg-1 and the reference gene GAPDH using the Light Cycler system (Roche), the SYBR Green master mix (Roche) and the primers shown in Table 2. Data was analysed using the LCS480 1.50.SP4 software (Roche). Culture medium was included as a background control, and results were represented as log-fold changes. Non-template controls were included in the assay.

### Total nitric oxide secretion

Total nitric oxide (NO) production in the culture supernatant was tested using a Nitrate Assay Kit (Beyotime, China) according to the manufacturer’s instructions. The treatment procedure was the same as for the qRT-PCR assay. At each time point, 100 μl of culture supernatant was collected per well and stored at −70 °C until detection. The absorbance was measured at 540 nm using a microplate reader (M2, Spectra Max) and the total NO concentration was calculated based on a standard curve, ranging from 2–80 mM/ml.

### Cell surface antigen expression

Expression of cell surface antigens CD80 and CD86 were determined by immunofluorescence staining and flow cytometry. PE mouse anti-mouse CD80 (BD Bioscences) and PE mouse anti-mouse CD86 (BD Bioscences) were used to stain cells stimulated by ZY-312 cell lysate (initial MOI = 500) for 3, 6, 12 h or by ZY-312 bacteria (MOI = 20, 100, 500) for 4 h. Expression of CD80 and CD86 was determined using a flow cytometer (BD, Accuri, C6) and analysed using the cflow PLUS software (version 1.0.264.15).

### Experimental replicates and statistical methods

All experiments were performed at least in triplicate using independent assays, and values were expressed as the mean ± standard error. Unpaired Student’s t-test was performed to determine statistically significant differences in phagocytic assays; one-way ANOVA test and Dunnett’s post-hoc test were performed to determine statistically significant differences in other experiments. A *p* value of <0.05 was considered to indicate statistical significance.

## Additional Information

**How to cite this article**: Deng, H. *et al*. A novel strain of *Bacteroides fragilis* enhances phagocytosis and polarises M1 macrophages. *Sci. Rep.* **6**, 29401; doi: 10.1038/srep29401 (2016).

## Figures and Tables

**Table 1:** 

(a)			
	pH 3.0 (SGF)	pH 4.0 (SGF)	pH 6.8 (SIF)
1 h	82.90 ± 8.74	94.54 ± 22.48	104.50 ± 20.35
2 h	95.06 ± 13.21	96.47 ± 23.93	102.17 ± 14.01
3 h	78.25 ± 18.88	93.72 ± 18.00	131.37 ± 34.06
**(b)**			
	**1% (bile)**	**2% (bile)**	**4% (bile)**
1 h	89.13 ± 18.2	111.65 ± 9.85	127.15 ± 33.52
2 h	101.3 ± 3.44	190.61 ± 15.54	126.80 ± 24.63
4 h	247.62 ± 17.73	364.40 ± 62.78	327.49 ± 23.20

Tolerance to SGF, SIF (**a**) and ox bile (**b**). (**a**) Survival rate after exposure to SGF at low pH and SIF for 1, 2, 3 h.

(**b**) Survival rate after exposure to 1%, 2%, 4% ox bile in TSB (10% FBS) for 1, 2, 4 h.

**Table 2:** Sequences of primers used in this study.

GAPDH-forward	5′-ATGACATCAAGAAGGTGGTGAAG-3′
GAPDH-reverse	5′-CCTGTTGCTGTAGCCGTATTC-3′
iNOS-forward	5′-CACCAAGCTGAACTTGAGCG-3′
iNOS-reverse	5′-CGTGGCTTTGGGCTCCTC-3′
Arg-1-forward	5′-TACMGACAGGGCTCCTTTCAG-3′
Arg-1-reverse	5′-AACCGAACGAAGCCTTGAGT-3′
IL-1β-forward	5′-ATGAAGTGCTCCTTCCAGGACCTG-3′
IL-1β-reverse	5′-CCTGGAGTGGAGAGCTTCAGTT-3′
IL-12-forward	5′-CACGGCAGCAGAATAAATA-3′
IL-12-reverse	5′-CTTGAGGGAGAAGTAGGAATG-3′
IL-10-forward	5′-GCTCTTACTGACTGGCATGAG-3′
IL-10-reverse	5′-CGCAGCTCTAGGAGCATGTG-3′

## References

[b1] Mazmanian S. K., Liu C. H., Tzianabos A. O. & Kasper D. L. An immunomodulatory molecule of symbiotic bacteria directs maturation of the host immune system. Cell 122, 107–118 (2005). 16009137 10.1016/j.cell.2005.05.007

[b2] Neish A. S. Microbes in gastrointestinal health and disease. Gastroenterology 136, 65–80 (2009). 19026645 10.1053/j.gastro.2008.10.080 PMC2892787

[b3] Wills-Karp M., Santeliz J. & Karp C. L. The germless theory of allergic disease: revisiting the hygiene hypothesis. Nat. Rev. Immunol. 1, 69–75 (2001). 11905816 10.1038/35095579

[b4] Kononen E., Jousimies-Somer H. & Asikainen S. Relationship between oral gram-negative anaerobic bacteria in saliva of the mother and the colonization of her edentulous infant. Oral. Microbiol. Immunol. 7, 273–276 (1992). 1494450 10.1111/j.1399-302x.1992.tb00587.x

[b5] Mazuski J. E. & Solomkin J. S. Intra-abdominal infections. Surg. Clin. North. Am. 89, 421–437 (2009). 19281892 10.1016/j.suc.2008.12.001

[b6] Mazmanian S. K. & Kasper D. L. The love-hate relationship between bacterial polysaccharides and the host immune system. Nat. Rev. Immunol. 6, 849–858 (2006). 17024229 10.1038/nri1956

[b7] Mazmanian S. K., Round J. L. & Kasper D. L. A microbial symbiosis factor prevents intestinal inflammatory disease. Nature 453, 620–625 (2008). 18509436 10.1038/nature07008

[b8] Hsiao E. Y. . Microbiota modulate behavioral and physiological abnormalities associated with neurodevelopmental disorders. Cell 155, 1451–1463 (2013). 24315484 10.1016/j.cell.2013.11.024 PMC3897394

[b9] Bloem K. . Interaction of the Capsular Polysaccharide A from *Bacteroides fragilis* with DC-SIGN on Human Dendritic Cells is Necessary for Its Processing and Presentation to T Cells. Front. Immunol. 4, 103 (2013). 23653626 10.3389/fimmu.2013.00103 PMC3644800

[b10] Cobb B. A., Wang Q., Tzianabos A. O. & Kasper D. L. Polysaccharide processing and presentation by the MHCII pathway. Cell 117, 677–687 (2004). 15163414 10.016/j.cell.2004.05.001 PMC2917993

[b11] Ortiz M. C. . Neisseria gonorrhoeae Modulates Immunity by Polarizing Human Macrophages to a M2 Profile. PLos One 10, e0130713 (2015). 26125939 10.1371/journal.pone.0130713 PMC4488386

[b12] Mills C. D. & Ley K. M1 and M2 macrophages: the chicken and the egg of immunity. J. Innate Immun. 6, 716–726 (2014). 25138714 10.1159/000364945 PMC4429858

[b13] Frankenberg T., Kirschnek S., Hacker H. & Hacker G. Phagocytosis-induced apoptosis of macrophages is linked to uptake, killing and degradation of bacteria. Eur. J. Immunol. 38, 204–215 (2008). 18085665 10.1002/eji.200737379

[b14] Mills C. D. Anatomy of a discovery: m1 and m2 macrophages. Front. Immunol. 6, 212 (2015). 25999950 10.3389/fimmu.2015.00212 PMC4419847

[b15] Biswas S. K. & Mantovani A. Macrophage plasticity and interaction with lymphocyte subsets: cancer as a paradigm. Nat. Immunol. 11, 889–896 (2010). 20856220 10.1038/ni.1937

[b16] Mills C. D., Kincaid K., Alt J. M., Heilman M. J. & Hill A. M. M-1/M-2 macrophages and the Th1/Th2 paradigm. J. Immunol. 164, 6166–6173 (2000).10843666

[b17] Sica A. & Mantovani A. Macrophage plasticity and polarization: *in vivo* veritas. J. Clin. Invest. 122, 787–795 (2012). 22378047 10.1172/JCI59643 PMC3287223

[b18] Ndamukong I. C., Gee J. & Smith C. J. The extracytoplasmic function sigma factor EcfO protects Bacteroides fragilis against oxidative stress. J. Bacteriol. 195, 145–155 (2013). 23104808 10.1128/JB.01491-12 PMC3536166

[b19] Pumbwe L. . Bile salts enhance bacterial co-aggregation, bacterial-intestinal epithelial cell adhesion, biofilm formation and antimicrobial resistance of Bacteroides fragilis. Microb. Pathog. 43, 78–87 (2007). 17524609 10.1016/j.micpath.2007.04.002

[b20] Dasgupta S., Erturk-Hasdemir D., Ochoa-Reparaz J., Reinecker H. C. & Kasper D. L. Plasmacytoid dendritic cells mediate anti-inflammatory responses to a gut commensal molecule via both innate and adaptive mechanisms. Cell Host Microbe 15, 413–423 (2014). 24721570 10.1016/j.chom.2014.03.006 PMC4020153

[b21] Wang Q. . A bacterial carbohydrate links innate and adaptive responses through Toll-like receptor 2. J. Exp. Med. 203, 2853–2863 (2006). 17178920 10.1084/jem.20062008 PMC2118167

[b22] Macatonia S. E. . Dendritic cells produce IL-12 and direct the development of Th1 cells from naive CD4+ T cells. J. Immunol. 154, 5071–5079 (1995). 7730613

[b23] Cobb B. A. & Kasper D. L. Zwitterionic capsular polysaccharides: the new MHCII-dependent antigens. Cell Microbiol. 7, 1398–1403 (2005). 16153240 10.1111/j.1462-5822.2005.00591.x

[b24] Stephen T. L. . Effect of B7-2 and CD40 signals from activated antigen-presenting cells on the ability of zwitterionic polysaccharides to induce T-Cell stimulation. Infect Immun. 73, 2184–2189 (2005). 15784561 10.1128/IAI.73.4.2184-2189.2005 PMC1087428

[b25] Van Gool S. W. . Blocking CD40 - CD154 and CD80/CD86 - CD28 interactions during primary allogeneic stimulation results in T cell anergy and high IL-10 production. Eur. J. Immunol. 29, 2367–2375 (1999). 10458748 10.1002/(SICI)1521-4141(199908)29:08<2367::AID-IMMU2367>3.0.CO;2-3

[b26] Cohen-Poradosu R., McLoughlin R. M., Lee J. C. & Kasper D. L. Bacteroides fragilis-stimulated interleukin-10 contains expanding disease. J. Infect. Dis. 204, 363–371 (2011). 21742834 10.1093/infdis/jir277

[b27] Round J. L. & Mazmanian S. K. Inducible Foxp3+ regulatory T-cell development by a commensal bacterium of the intestinal microbiota. Proc. Natl. Acad. Sci. USA 107, 12204–12209 (2010). 20566854 10.1073/pnas.0909122107 PMC2901479

[b28] Zhang Z. . Isolation and identification of quercetin degrading bacteria from human fecal microbes. PLos One 9, e90531 (2014). 24594786 10.1371/journal.pone.0090531 PMC3942438

[b29] Sim I. . *In vitro* assessment of the gastrointestinal tolerance and immunomodulatory function of Bacillus methylotrophicus isolated from a traditional Korean fermented soybean food. J. Appl. Microbiol. 118, 718–726 (2015). 25494714 10.1111/jam.12719

[b30] Charteris W. P., Kelly P. M., Morelli L. & Collins J. K. Development and application of an *in vitro* methodology to determine the transit tolerance of potentially probiotic Lactobacillus and Bifidobacterium species in the upper human gastrointestinal tract. J. Appl. Microbiol. 84, 759–768 (1998). 9674129 10.1046/j.1365-2672.1998.00407.x

[b31] Brown K. L. . Host defense peptide LL-37 selectively reduces proinflammatory macrophage responses. J. Immunol. 186, 5497–5505 (2011). 21441450 10.4049/jimmunol.1002508

[b32] Mohawk K. L., Melton-Celsa A. R., Zangari T., Carroll E. E. & O’Brien A. D. Pathogenesis of Escherichia coli O157:H7 strain 86-24 following oral infection of BALB/c mice with an intact commensal flora. Microb. Pathog. 48, 131–142 (2010). 20096770 10.1016/j.micpath.2010.01.003 PMC2834854

[b33] Roman L. . The *in vitro* effect of probiotic Vagococcus fluvialis on the innate immune parameters of Sparus aurata and Dicentrarchus labrax. Fish. Shellfish. Immunol. 33, 1071–1075 (2012). 22864109 10.1016/j.fsi.2012.06.028

